# Report of a Case of Ulceration of the Cervix Uteri, Read before the Medical Association of Georgia

**Published:** 1860-05

**Authors:** A. M. Boyd

**Affiliations:** Cave Spring, Georgia


### ARTICLE IV.

*Report of a Case of Ulceration of the Cervix Uteri, read before the Medical Association of Georgia.* By A. M. Boyd, M. D., of Cave Spring, Georgia.

In giving the history and treatment of the following case, it is deemed wholly unnecessary to indulge in a lengthy article on the pathology of uterine inflammation and ulceration, so much more having already been written than has been or ever will be reduced to practice.

The object is to give a simple statement of the case as it occurred, to show the unsatisfactory results of the old system of treating such disorders, and if possible, to furnish facts for the benefit of the profession.

Mrs. S——, aged about 25 years, had been suffering from what herself and physicians, *termed*, *falling of the womb.* Her own statement of her case was that, at about fifteen, her menstrual function was established, at first, healthy, but in two or three days was arrested by a severe cold; from then until she was 18, her menstrual periods were very irregular, sometimes not opening at all, at others the flow was and nearly colorless.

Her health, from the first, rapidly declined; she applied for medical aid, and was treated with tonics, vaginal injections, &c., but obtained no relief. At 18, three years after the uterine disturbance, she was married, and the first recurrence of her menstrua after this event, was about as when the function was first established, except that it was not arrested by cold; and from that time until I saw her, (Dec. 5th, 1857,) she states that there was a total suspension of her catamenia; she had consulted physician after physician, all of whom she said, pronounced her case falling of the womb, and treated her accordingly. Despairing of a cure from the physicians, she betook herself to the *invalids curse*—that most deadly foe to the human health—quacks and quack nostrums, until scarcely able to sit up, a mere skeleton, if I may be allowed the expression, and was at the time, under the treatment of a celebrated uterine quack. I found she was suffering from severe pelvic pain, and weighty pains in the thighs and in the lumbar region, with a profuse leucorrhœa; her digestive function was almost suspended—the stomach rejecting nearly everything taken into it. Her lungs were also considerably involved, coughed almost constantly.

After receiving the history of the case, and looking well to her present condition, I came to the conclusion that it was almost hopeless. She weighed about eighty pounds.

I decided to withhold any opinion for the present, and directed her to leave off the use of medicine entirely, until I returned. I could promise her nothing until I fully satisfied myself as to the extent of her womb disease.

On the 7th, I found her up; she stated that, she felt better than she had for a month; she was from under the influence of disgusting nostrums. An examination of her vagina revealed a hard, swollen and extremely sensitive cervix, resting on the perineum; the ostince partially dilated, though not sufficient to admit the index finger. The object of this examination was not for the purpose of diagnosis proper, but to ascertain the situation or rather location of the cervix.

I then introduced a medium size cylindrical glass speculum and found several ulcers around and in the ostince, to each of which was applied solid nitrate of silver, with a common speculum forceps. The caustic was also passed as far into the cervical cavity as convenient, without giving too much pain—directed an injection of two or three pints of cold water to prevent the dissolved caustic from excoriating the mucous membrane of the vagina which was in a healthy condition. She then used an injection of equal parts of sweet milk and warm water, three times per day, until the eschars came off which is usually in two or three days. In almost every instance this is attended with a slight hemorrhage, which for a time relieves the ingorged cervix, and the pain and weight in the pelvis and loins.

After this had taken place, and until the next application was made, (which is usually in six days,) the patient made use of an injection of zinci sulphas and plumbi acetas, aa x grs.; water, ℥ viii, mix and inject three times per day.

The following pill was prescribed as a tonic:

Iron by hydrogen, ℈ii.

Sulphate quinine, ℈i.

Extract gentian, *Q. S.*

Make 20 pills and give one three times per day, with anodyne cough mixture to be taken only as the cough was troublesome.

I decided to give her the benefit of this course of treatment so highly recommended by Dr. James Henry Bennett, of London, all others having proven only palliative, and in the end a total failure. Success in this plan of treatment depends upon the application of solid nitrate of silver to the ulcerating surface, which should be repeated at intervals of from five to eight days, except during the menstrual period, with such tonic remedies as are best suited to the state of the patient's general health.

The formula above, seems admirably suited to the majority of cases, from the fact that chlorosis or anemia nearly always co-existed with the malady in question.

13th. Found patient's condition improved; cough not so troublesome; appetite better, and could retain her food; the eschars had passed off, attended with the usual amount of hemorrhage, with considerable relief. The ulcers presented a little more healthy appearance, but no perceptible change in the size of cervix. Made the same application and continued the treatment.

24th. Symptoms all improved; ulcers begin to present points of granulation, except in the ostince. Applied the caustic as before, passing it well up into the cervical cavity. Left off the injection of zinc and lead, and used a saturated solution of chlorate potash.

31st. I was surprised at finding my patient so much altered in appearance—she seems cheerful, and thinks she will get well. Her appetite is much improved, and digestion comparatively good. Leucorrhœa disappeared entirely; ulcers cicatrized, except in the cervical cavity, which looks healthy; the neck of the womb much reduced in size, soft, and presents a pink hue, as in the healthy organ—applied caustic and continued the treatment.

January 7th, 1858. Mrs. S—— seems so much improved that I begin to entertain hopes of her final recovery. On examination I found the cervix high up in the vagina, much reduced in size, soft and healthy. Thought it better, however, to apply the caustic again to the cavity, which was pretty firmly closed, somewhat inflamed, but not ulcerating, for the first time during the treatment. The patient complained of acute pain when the caustic passed into the cavity.

As yet, there has been no symptoms of her menses, except at times a few wandering pains as in dysmenorrhœa.—Continued the injection of chlorate potash, and prescribed instead of tonic pills, balsam fir, ℥ i, French brandy, ℥ xii, shake well together, and take a tablespoonful three times per day.

February 15th. Since January 7th, Mrs. S—— has been rapidly improving—all her pelvic weight and pain has disappeared; her breasts begin to enlarge and she seems as if she had conceived—still her menses are absent. The ulcers all cicatrized—cervix high up and of normal size, soft and healthy. Continued balsam fir, with injections of cold water, daily. In March her menses appeared in a healthy condition, coming on, she says, without pain, and continued six days—quantity about normal—all the symptoms of pregnancy disappeared. Her menstrua recurred regularly each month, unattended with pain; and her weight had increased from 80 to 112 pounds.

In December following, her menses disappeared altogether, and she presented all the symptoms of being pregnant. I saw her in May 1859—she was attacked with incontinence of urine, for which I prescribed infusion uva ursi, and in a few days she was entirely relieved. With this exception she passed through her gestating period without difficulty, and on the 30th of August she gave birth to a healthy female child, weighing 6½ pounds.

Her accouchment was short and easy, and she had a good getting up, since which time she has enjoyed better health than she ever did before.

What seems most remarkable in this case is, the rapidity with which it yielded to treatment, after having been so deeply rooted in the system, and the length of time it had existed. I have since treated several cases with equal success, though none of so long standing, yet sufficiently aggravated to test the merits of the system. If properly carried out both by physician and patient, we venture the assertion that, the majority of suffering females might not only be relieved, but many of them permanently cured.

